# Supplementary material for: A 3D-Printed Aqueous Drainage Tube with an Expandable Inner Diameter to Accommodate the Intraocular Pressure (IOP) Fluctuations After Glaucoma Surgery
Source: Polymers (Basel). 2025 Jan 5;17(1):118. doi: 10.3390/polym17010118 (PMC11722927; doi:10.3390/polym17010118)
Supplement: Supplementary file 1 [file polymers-17-00118-s001.zip › polymers-3365030-supplementary.pdf]

Supplementary data

A 3D-Printed Aqueous Drainage Tube with an Expandable Inner Diameter to Accommodate the Intraocular Pressure (IOP) Fluctuations After Glaucoma Surgery

*Jae-Seok Kim [1], Hun-Jin Jeong [2], Ji-Woo Park [3], So-Jung Gwak [3,4], Jeong-Sun Han [5], Kyoung In Jung [5] and Seung-Jae Lee [1,4,6]**

1 Department of Mechanical Engineering, Wonkwang University, 460 Iksandae-ro, Iksan 54538, Republic of Korea; jaeseokkim311@gmail.com

2 Regenerative Engineering Laboratory, Columbia University, 630W 168th ST, New York, 10032, USA; hj2607@cumc.columbia.edu

3 Department of Chemical Engineering, Wonkwang University, 460 Iksandae-ro, Iksan 54538, Republic of Korea; wldn0106@naver.com (J.-W.P.)

4 MECHABIO Group, Wonkwang University, 460 Iksandae-ro, Iksan 54538, Jeonbuk, Republic of Korea

5 Department of Ophthalmology, Seoul St. Mary's Hospital, College of Medicine, The Catholic University of Korea, Seoul 06591, Republic of Korea; winehan@catholic.ac.kr (J.-S.H.); ezilean@hanmail.net (K.I.J.)

6 Division of Mechanical Engineering, Wonkwang University, 460 Iksandae-ro, Iksan 54538, Republic of Korea

* Correspondence: sjlee411@wku.ac.kr

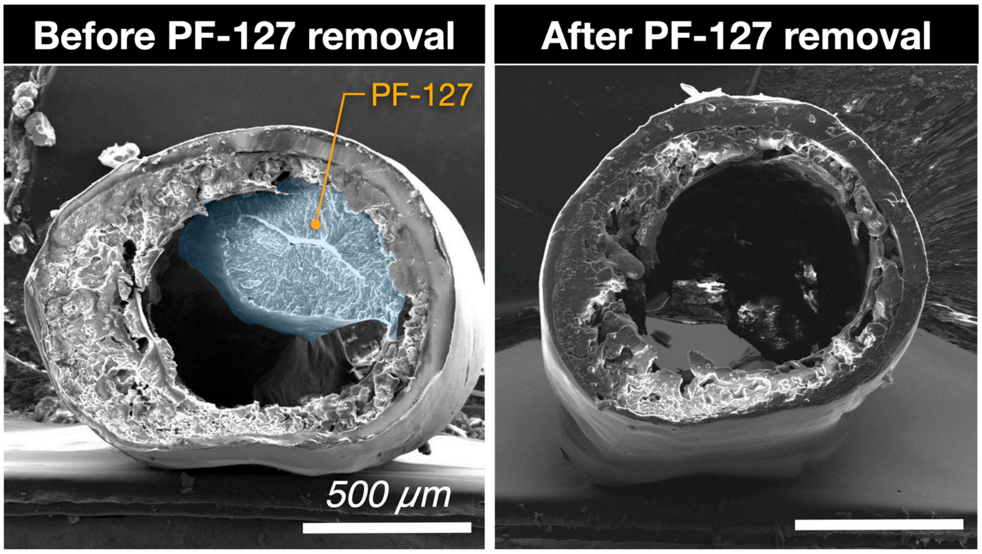

Supplementary figure 1. SEM image before and after removal of PF-127. (Blueish highlight indicated PF-127 at the core)

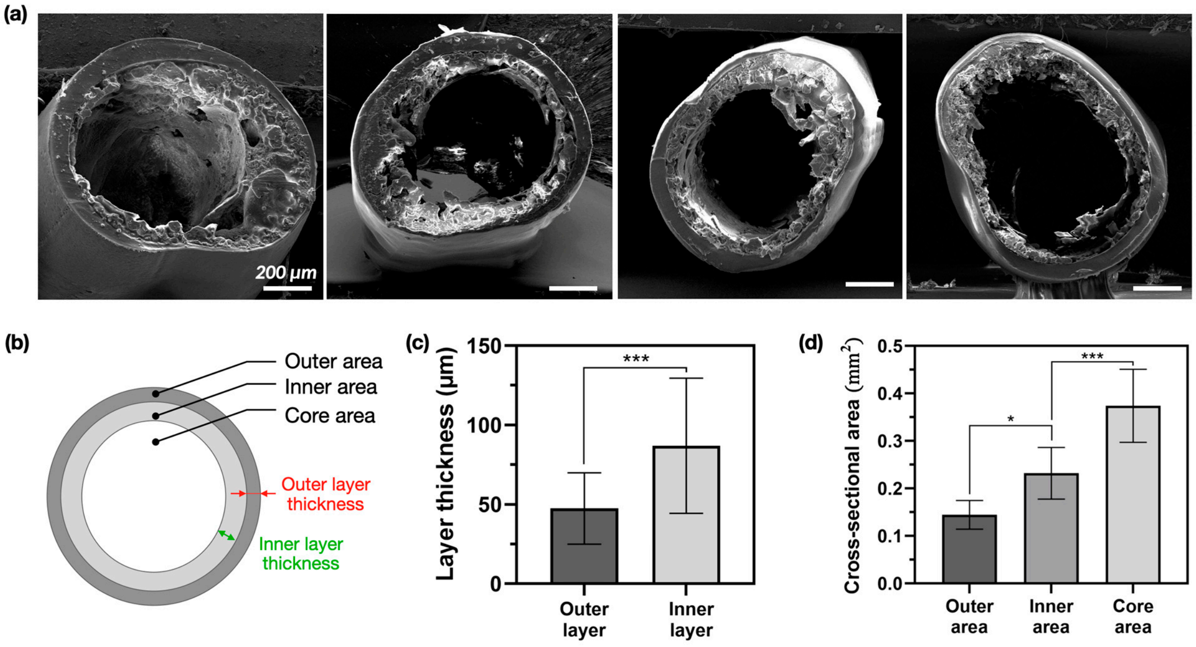

Supplementary figure 2. Analysis of fabricated aqueous drainage tube: **(a)** scanning electron microscopy image of the aqueous drainage tube; **(b)** schematic of cross-sectional view; **(c)** layer thickness measurement results; **(d)** cross-sectional area measurement results (*** = $p \leq 0.001$, * = $p \leq 0.05$).

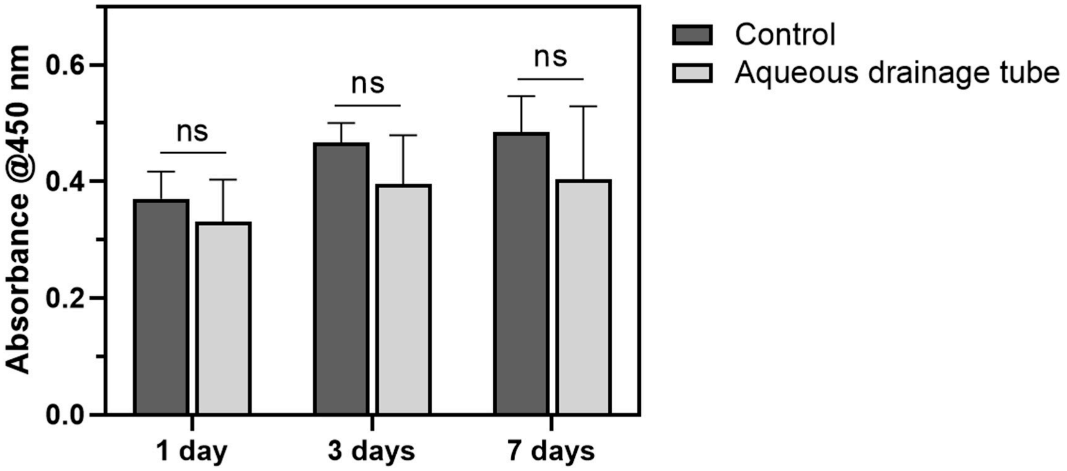

Supplementary figure 3. CCK-8 analysis result of the fabricated aqueous drainage tube at 1, 3, and 7 days (ns = no significant difference, $p > 0.05$).
